# Low linkage disequilibrium in wild *Anopheles gambiae s.l*. populations

**DOI:** 10.1186/1471-2156-11-81

**Published:** 2010-09-15

**Authors:** Caroline Harris, François Rousset, Isabelle Morlais, Didier Fontenille, Anna Cohuet

**Affiliations:** 1 Laboratoire de Lutte Contre les Insectes Nuisibles, Unité de Recherche 016-Institut de Recherche pour le Développement, B.P. 64501, 34394 Montpellier Cedex 5, France; 2 Institut des Sciences de l'Évolution, Université Montpellier 2, CNRS, Place Eugène Bataillon, CC065, Montpellier, Cedex 5, France; 3 Institut de Recherche pour le Développement, Institut de Recherche en Sciences de la Santé, Bobo Dioulasso, Burkina Faso

## Abstract

**Background:**

In the malaria vector *Anopheles gambiae*, understanding diversity in natural populations and genetic components of important phenotypes such as resistance to malaria infection is crucial for developing new malaria transmission blocking strategies. The design and interpretation of many studies here depends critically on Linkage disequilibrium (LD). For example in association studies, LD determines the density of Single Nucleotide Polymorphisms (SNPs) to be genotyped to represent the majority of the genomic information. Here, we aim to determine LD in wild *An. gambiae s.l*. populations in 4 genes potentially involved in mosquito immune responses against pathogens (*Gambicin*, *NOS*, *REL2 *and *FBN9*) using previously published and newly generated sequences.

**Results:**

The level of LD between SNP pairs in cloned sequences of each gene was determined for 7 species (or incipient species) of the *An. gambiae *complex. In all tested genes and species, LD between SNPs was low: even at short distances (< 200 bp), most SNP pairs gave an r^2 ^< 0.3. Mean r^2 ^ranged from 0.073 to 0.766. In most genes and species LD decayed very rapidly with increasing inter-marker distance.

**Conclusions:**

These results are of great interest for the development of large scale polymorphism studies, as LD generally falls below any useful limit. It indicates that very fine scale SNP detection will be required to give an overall view of genome-wide polymorphism. Perhaps a more feasible approach to genome wide association studies is to use targeted approaches using candidate gene selection to detect association to phenotypes of interest.

## Background

When alleles at different loci appear together in individuals more often than would be expected by chance they are said to be in Linkage Disequilibrium (LD) [1]. LD is an indicator of the rate of recombination events between markers during meiosis. In addition to nucleotide distance, the effective recombination rate can be affected by numerous forces in natural populations such as selection that maintains certain allele associations (epistasis), genetic drift, population structure and demographic changes. Non random association between variants has recently become the focus of intense study in the hope that it might facilitate the mapping of complex trait loci through genome wide association studies (GWAS). Indeed, recent progress in the technological ability to genotype genetic variation [2] opens promising possibilities for identification of variants linked to phenotypes of interest. However, the ability to detect association critically depends on the extent of LD between causative alleles and surrounding markers. When LD extends over large genomic regions, there is a higher chance of finding association with the drawback that the potentially long physical distance between the gene of interest and an associated marker can make causative gene identification tedious. On the other hand, limited LD requires a much denser marker map to find associations, but, when found, identifying the causative allele is expected to be more straightforward [3].

The *Anopheles gambiae *species complex is of great interest due to its substantial role in malaria transmission. The two molecular forms or incipient species, M and S, of *Anopheles gambiae s.s*. and *Anopheles arabiensis *are major vectors throughout sub Saharan Africa. The other species of the complex can have high local importance in malaria transmission or more minor roles depending on their biology and distribution [4]. Due to its epidemiological importance, several genomes [5,6] and genes of interest [e.g. [7-10]] have been sequenced in *An. gambiae s.s*., providing a large data set of SNPs (Single Nucleotide Polymorphisms). Many of these variants are shared with the other members of the complex [7,8]. Today, the development of large scale genotyping tools makes the implementation of GWAS in *An. gambiae s.s*. and possibly the other members very realistic. However, to date little is known about LD in natural populations. In *An. gambiae s.s*., analysis of SNPs in six genes located in and around the 2La chromosomal inversion revealed LD over more than 30 Mbp [11]. Strong LD was also detected within and between centromeric regions of the two incipient species M and S of *An. gambiae s.s.*[12]. This LD likely indicated subdivided populations adapted to different environments or incipient species, indeed, limited recombination rates in these chromosomal areas are hypothesized to be involved in environmental adaptation by maintaining combinations of alleles adapted to given conditions [13] and/or implicated in the speciation process through cause or consequence [12]. In other regions of the genome, very little data is available to our knowledge in natural populations; LD was measured only to exclude redundancy of markers or Wahlund effect in population genetic studies and rarely showed linked markers [e.g. [14-16]]. For instance, Lehmann *et al. *[16] tested the LD between microsatellite markers in several populations and observed only few pairwise combinations in significant LD, corresponding to the proportion of the tests expected to be significant by chance alone. This indicated random association between the tested markers spread throughout the genome of *An. gambiae s.s*., however this is only mildly informative for short distance LD and the density of markers to be used in association studies.

In this study, we aimed to determine LD in *An. gambiae s.l*. between short range markers. LD data will be particularly informative for mapping genes involved in susceptibility to infection. In this context, immune related genes are primary candidates and rates of LD decay in these genes will be crucial in determining the density of markers to be used in such association studies. Four immune related genes were selected, namely, *Gambicin*, *NOS*, *REL2 *and *FBN9*, representative of different functions in the immune response and located in different chromosomal regions. *Gambicin *codes for an important antimicrobial peptide, which currently has no known specificity to *Plasmodium *[17]. *NOS *codes for Nitric oxide synthase, which markedly controls the infection level of *Plasmodium *in *Anopheles *[18,19] but is likely to play different roles depending on parasite species [20]. *REL2 *is an NF-KappaB-like transcription factor that affects the development of *Plasmodium *in *Anopheles *in a conserved manner across several species of parasite and mosquito [21]. *FBN9 *codes for a Fibrinogen-domain protein whose silencing increases *P. falciparum *development [17].

For each gene, sequences of seven species of the *An. gambiae *complex were analyzed and LD between polymorphic sites measured to estimate the extent of information given by genotyping a single polymorphism. We used previously published sequences [8] for 6 of the species and produced new data for a population of *An. gambiae s.s *M molecular form from Cameroon. Phased sequences were used to provide accurate haplotype data for estimating LD. To our knowledge, this is the first study on LD in *An. gambiae *based on phased sequences, allowing powerful analysis of LD decay over short distances. Moreover it is informative across almost all species members of the *An. gambiae *complex and focuses on genes of interest for future association studies.

## Methods

Phased sequences, resulting from cloned DNA, were previously published for the genes *Gambicin *(AGAP008645), *NOS *(AGAP008255), *REL2 *(AGAP006747) and *FBN9 *(AGAP011197) for 5 to 14 field collected individuals of *An. gambiae *S form, *An. arabiensis*, *An. melas*, *An. merus*, *An. quadriannulatus *A and *An. bwambae *[8]. No evidence for positive selection was identified in these genes. Here, we provide data for the corresponding gene fragments for 16 individuals of *An. gambiae *M form collected in Simbock in South Cameroon.

Genes were localized on the *An. gambiae s.s*. genome using Vectorbase [20], and their positions relative to polymorphic chromosomal inversions determined [22]. *Gambicin *is positioned in subdivision 31A on chromosome 3R, where the inversions 3Ra in *An. arabiensis *and 3Re in *An. melas *are known to be polymorphic. *NOS *is in the subdivision 30A on chromosome 3R, it is located in polymorphic inversions 3Ra in *An. arabiensis*, 3Rb in *An. bwambae *and 3Re in *An. melas*. *FBN9 *is in subdivision 42A on chromosome 3L, where no polymorphic inversions are known within each species. *REL2 *is in subdivision 25D on chromosome 2L, here only the rare inversion 2Ld is known to be polymorphic in *An. arabiensis *but was not observed in Cameroon where the specimens were collected (Frederic Simard, personal communication).

To sequence *An. gambiae *M form individuals, DNA was extracted from mosquitoes as previously described [9]. Species and molecular forms were determined by diagnostic PCR [23]. Gene amplifications were carried out using the external primers and conditions previously published [8]. PCR products were cloned using the TOPO TA Cloning^® ^Kit for Sequencing (Invitrogen) and a minimum of five transformed colonies selected for sequencing. Inserts were amplified by PCR from the plasmid using the same external primers/conditions and were sequenced in both directions using the Big Dye Terminator v3.1 Sequencing Kit (Applied Biosystems). Sequences were verified by eye in SeqScape (Applied Biosystems) and aligned in Mega v.4.0.2 [24]. High Fidelity Taq was used (Platinum^® ^Taq DNA Polymerase High Fidelity, Invitrogen) in all PCRs to limit miss-incorporations. Sequences have been submitted to GenBank under accession numbers GU990095 to GU990222.

Analyses of polymorphism were carried out on all the previously published and new *An. gambiae *M form sequences using DNAsp v.5.10 [25]. LD was measured as r for each pair of SNPs in each gene and species, significance (P < 0.05) was tested using Fisher's exact test in DNAsp and the Bonferroni procedure applied to correct for multiple testing. r was converted to r^2 ^and graphs of r^2 ^relative to the distance between pairs of polymorphic sites plotted in R v.2.10.0 [26]. LD decay lines were modeled again in R by fitting data to the expectations of a simple population genetic model [27] using the non-linear least squares method, and to a nonparametric model. The non-linear fit to expectations failed (nls function in R failed to converge) for some species/locus combinations because the observed patterns deviated too much from the expectations, in particular, due to them showing an increase in LD at short distances, or, LD measures that exceed the maximum allowed by the analytical expression [[27] p.77]. Hence, only the results of the nonparametric model will be presented. The non-parametric model was a "generalized additive model" where the fit is a linear combination of observed values, whose coefficients are given by a cubic spline and the degree of smoothing determined by generalized cross-validation [28]. This computation was performed using the gam function from the mgcv package in R.

Moreover, in order to detect LD haploblocks, grid plots were generated using Haploview 4.2 [29] for the *An. gambiae *M sequences for each of the four tested genes.

To calculate whether there are significant differences in LD between genes and species, mean values of the r^2 ^estimates were considered in each locus and species. The 7 values per locus (corresponding to the 7 species) or 4 values per species (corresponding to the 4 loci) were listed and compared by the Wilcoxon test by pair of loci or pair of species. As the numbers of values included in the tests were critically low, we attempted to increase the power of the tests by generating 4 values per locus and species. For that purpose, the sequences were cut into 4 segments of equal length and mean r^2 ^calculated for each segment to give independent values (4 mean values for one gene in one species). The 4 mean r^2 ^values were listed and grouped either according to gene (4 values for each of the 7 species resulting in 28 values in each list) or species (4 values for each of the 4 loci resulting in 16 values in each list) and the Wilcoxon test calculated in R to look for significant differences between groups (either each gene or each species).

## Results and discussion

**Figure 1:**
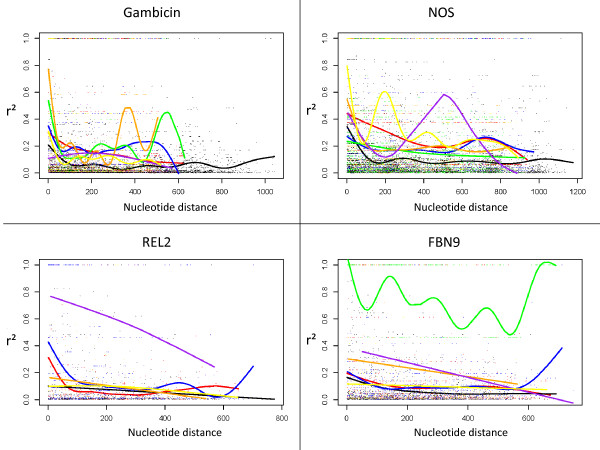
**Plots of LD decay over distance for 4 immunity genes in *An. gambiae s.l.***. Each data point represents a pairwise measure of r^2 ^as a function of nucleotide distance (bases). *An. gambiae *M form (black), *An. gambiae *S form (blue), *An. arabiensis *(red), *An. bwambae *(green), *An. melas *(purple), *An. merus *(orange) and *An. quadriannulatus *(yellow). LD decay curves are fitted by the gam function in the mgcv package, using default options.

**Figure 2:**
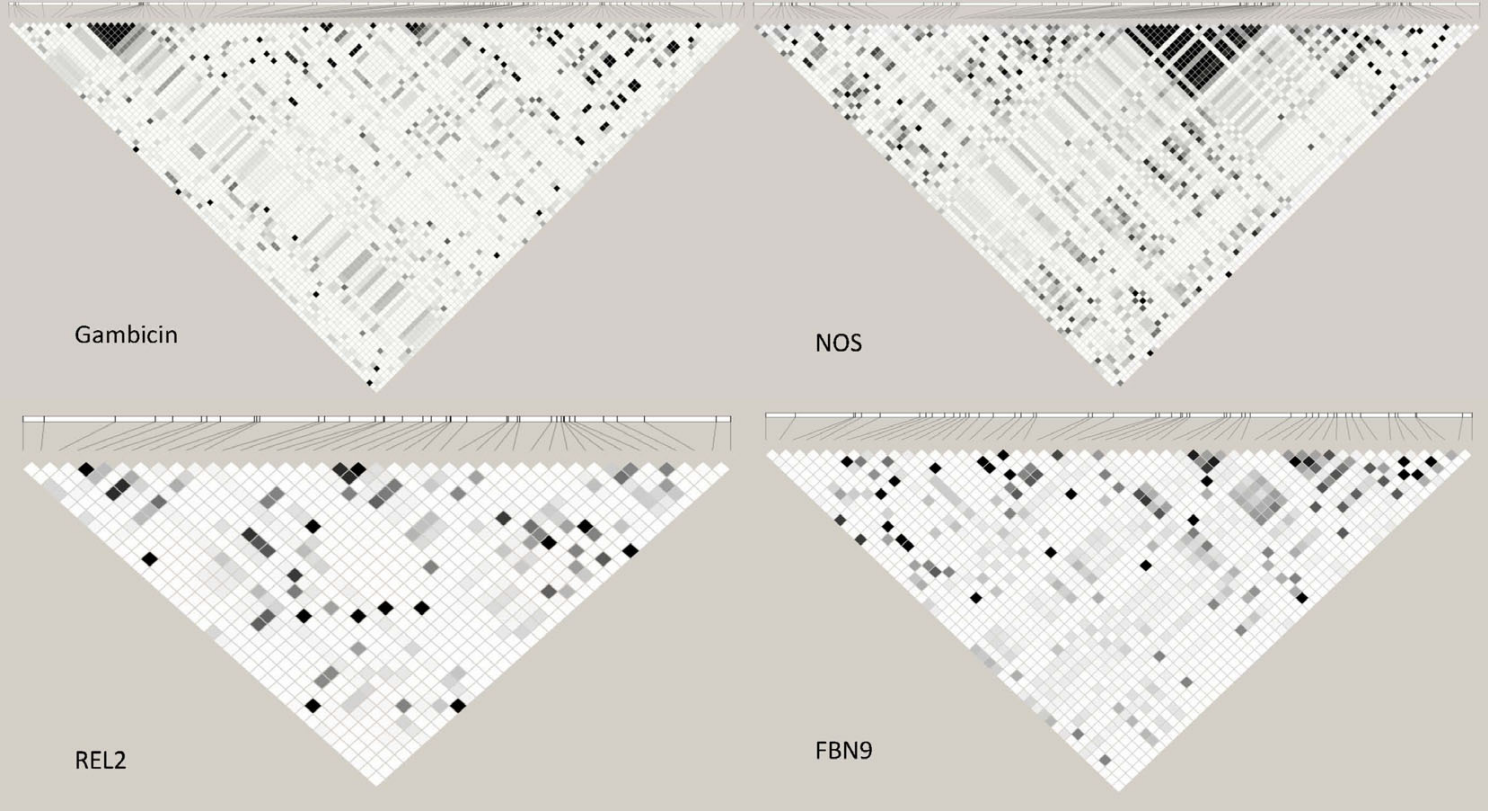
**Grid plots for 4 immunity genes in *An. gambiae *M form**. Each square corresponds to one LD value (r^2^) between 2 variant sites using a color code from a complete lack of LD (r^2 ^= 0: white) to a perfect LD (r^2 ^= 1: black). Relative position of the SNPs is represented on the top line.

Sequences for 16 individuals of the *An. gambiae *M form were analyzed for the four genes. The polymorphism parameters are given in Table 1. Genetic diversity (Pi) in *An. gambiae *M form, ranging from 0.0099 to 0.0253, has comparable values to other species of the complex [8] and to other immunity genes [30-32]. As expected, the inclusion of introns increases the diversity compared to studies including only coding regions [7]. The high number of variant sites in *An. gambiae *M form and other species allowed a large number of pairwise measures of LD in almost all species and tested genes (Table 2). A very small proportion of pairwise measures was significantly in LD, although *An. gambiae *M revealed slightly more significant tests than other species, most likely a consequence of the bigger sample size (Table 2). Only *REL2 *in *An. bwambae *was not polymorphic. Plots of r^2 ^as a function of nucleotide distance are presented in Figure 1 for all species and genes and grid plots for *An. gambiae *M in Figure 2. Mean values of r^2 ^(using whole sequences) for each gene and species are shown in Table 2.

**Table 1:** Sequence and polymorphism parameters of the four amplified immunity genes in *An. gambiae *M form

Gene	Number of individuals	Length of sequences analyzed (bp)	Number of haplotypes	Number of polymorphic sites	Nucleotide diversity (Pi)		
					
					Total	Synonymous	**Non- syn**.
*Gambicin*	16	1108	24	105	0.0253	0.0155	0.0032
*NOS*	16	1238	19	99	0.0247	0.0383	0.0009
*REL2*	16	834	21	40	0.0099	0.0220	0.0039
*FBN9*	16	735	18	53	0.0159	0.0664	0.0021

**Table 2:** Number of pairwise measures of r^2 ^and number of significant pairwise measures using the Fisher's exact test after Bonferroni correction in brackets (all in bold) and mean r^2 ^(standard deviation in brackets)

Gene	*An. gambiae *M form	*An. gambiae *S form	*An. arabiensis*	*An. bwambae*	*An. melas*	*An. merus*	*An. quadriannulatus*
*Gambicin*	**n = 16**	**n = 5**	**n = 6**	**n = 6**	**n = 6**	**n = 5**	**n = 5**
							
	**4465 (34)** 0.073 (SD 0.166)	**630 (0)** 0.192 (SD 0.270)	**1770 (0)** 0.166 (SD 0.311)	**253 (0)** 0.194 (SD 0.317)	**253 (0)** 0.121 (SD 0.270)	**378 (0)** 0.299 (SD 0.344)	**741 (0)** 0.166 (SD 0.244)
							
*NOS*	**n = 16**	**n = 6**	**n = 5**	**n = 7**	**n = 5**	**n = 6**	**n = 5**
							
	**4371 (141)** 0.200 (SD 0.206)	**1326 (0)** 0.198 (SD 0.274)	**820 (0)** 0.282 (SD 0.295)	**1770 (0)** 0.172 (SD 0.247)	**55 (0)** 0.243 (SD 0.292)	**990 (0)** 0.246 (SD 0.289)	**351 (0)** 0.356 (SD 0.330)
							
*REL2*	**n = 16**	**n = 14**	**n = 10**	**n = 6**	**n = 8**	**n = 7**	**n = 8**
							
	**703 (9)** 0.073 (SD 0.175)	**703 (11)** 0.143 (SD 0.264)	**378 (2)** 0.081 (SD 0.172)	**0 (0)** NA	**21 (0)** 0.577 (SD 0.415)	**55 (0)** 0.115 (SD 0.149)	**55 (0)** 0.091 (SD 0.207)
							
*FBN9*	**n = 16**	**n = 7**	**n = 7**	**n = 7**	**n = 7**	**n = 7**	**n = 7**
							
	**1378 (16)** 0.078 (SD 0.175)	**780 (0)** 0.106 (SD 0.156)	**1081(0)** 0.108 (SD 0.206)	**190 (0)** 0.766 (SD 0.285)	**15 (0)** 0.203 (SD 0.264)	**66 (0)** 0.231 (SD 0.364)	**276 (0)** 0.103 (SD 0.192)

NA: no available data when sequences are not polymorphic. n = number of individuals.

Mean r^2 ^values in genes and species range from 0.073 to 0.766, with most falling below 0.3. Only three mean values exceed this threshold; they correspond to *NOS *in *An. quadriannulatus*, *REL2 *in *An. melas *and *FBN9 *in *An. bwambae*. These genes are not positioned in polymorphic chromosomal inversions in these species suggesting that the higher values of r^2 ^are not the result of limited recombination rates due to chromosomal arrangements. All other mean values are below 0.3, which reveals very limited LD taking into account the short distance between polymorphic sites (maximum distance between SNPs is 1176 bases). Indeed, compared to other species, the LD values observed here are among the lowest. Similar low LD levels were previously observed, mainly in plants [33-36]. Much higher LD is commonly observed in a wide range of species, for example cultivated and wild plants [37,38], birds [3] and mammals [39-43]. In *Drosophila*, LD often appeared to be higher than what we observed in *Anopheles*, but was also very variable depending on gene and genomic region [44-46] probably as a result of variable recombination rates and natural selection [47]. In our data, the comparison of mean r^2 ^values in full sequences and sequence fragments revealed no significant differences between species or genes. This suggests that in different genomic regions, and members of the *An. gambiae *complex, LD in immunity genes, and probably others, is limited. However the small number of genes and chromosomal regions tested in the present study cannot allow conclusion for the whole *An. gambiae *genome as variations along the chromosomes and between mosquito populations are expected. In particular, the effect of chromosomal inversions was not tested here. The populations of *An. gambiae s.s*. (M and S) from Cameroon are known to be almost fixed for the standard chromosomal arrangements [48]. Testing the effect of the major chromosomal inversions of *An. gambiae s.s*. on LD would require sampling of populations polymorphic for these inversions, karyotyping the chromosomes and sequencing numerous genes inside and outside the inversions. Such a study would be of high interest as the resolution necessary for association studies could vary in chromosomal inversions or other genomic regions, and important genes involved in adaptation or controlling *Plasmodium *infection are expected to be located inside chromosomal inversions [22,49].

LD curves in *An. gambiae *species show very fast decay for most of the tested genes and species. Generally, at very short nucleotide distances, less than 200 bases, LD decay curves were below an r^2 ^of 0.3, although sporadic LD peaks were observed over distance. In exception to this is *An. bwambae FBN9 *whose decay curve never falls as low as r^2 ^= 0.3 in the 807 base region tested (Figure 1). The grid plots of *An. gambiae *M form showed similar results with few pairwise estimates in LD. Interestingly some haploblocks were observed (in *Gambicin *and *NOS*) showing strong LD between markers. This pattern was observed only for very short distance markers, not more distant than 50 base pairs (Figure 2). This suggested variation in LD along the genes but a very limited extension of high LD blocks. In association studies, depending on the contribution of the causative allele on the observed phenotype and the sample size, a minimum threshold of r^2 ^≥ 0.33 to 0.8 can be used to consider whether SNPs above this limit with the causative SNP are potentially indicative of association [47,50]. This suggests that, for GWAS in *An. gambiae s.l*., very dense marker coverage will be required and the development of high-throughput genotyping tools essential for whole genome scans. However, in candidate gene association studies, a very high resolution genetic map can be more feasible by limiting genotyping to genes that have functional relevance. Also a rapid breakdown of LD will be favorable for identification of causative genes located in quantitative trait loci (QTL) by favoring high resolution mapping.

LD in a population is the result of various parameters, for example recombination and mutation rates, population structure and demographic history [47]. In species of the *An. gambiae *complex, their demographic history has been closely linked to anthropic changes [51] and their population sizes probably drastically increased some thousands of years ago. Rapid population growth decreases LD, and could be one of the reasons for the limited LD we observe here but could also be in part due to high mutation and recombination rates.

## Conclusion

In this study, we observed limited LD in wild populations of seven species of the *An. gambiae *complex in four immunity genes. This suggests that GWAS will require a huge effort in genotyping and that a more realistic approach might be to search for functional variations in putative candidate loci. On the other hand, the rapid decay of LD suggests that it will be possible to map functional variation at very fine scales in *An. gambiae s.l*. populations. The present study however involved a limited number of chromosomal regions and populations so further work is needed to ascertain LD throughout *An. gambiae *genomes.

## Authors' contributions

A.C., I.M., F.R. and D.F. designed the study; C.H. carried out the experiments; A.C., C.H. and F.R. analysed the data; and A.C., C.H., F.R., I.M. and D.F. wrote the paper. All authors read and approved the final manuscript.
